# From Conventional Therapy to Precision Medicine in Inflammatory Bowel Disease: A State-of-the-Art Review

**DOI:** 10.3390/biomedicines14040798

**Published:** 2026-04-01

**Authors:** Anwar Almajdi, Mohammad Shehab

**Affiliations:** 1 Clinical Pharmacy Unit, Pharmacy Department, Al Jahra Hospital, Ministry of Health, Aljahra 03200, Kuwait; a.almajdi@moh.gov.kw; 2 Program of Medicine, College of Medicine and Health Sciences, Abdullah Al Salem University, Khaldiya 42167, Kuwait; 3 Division of Gastroenterology and Hepatology, McGill University Health Centre, Montreal, QC H3G 2M, Canada; 4 Division of Gastroenterology, Department of Internal Medicine, Mubarak Al-Kabeer University Hospital, Kuwait City 47060, Kuwait

**Keywords:** IBD, personalized medicine, multi-omics, biologics, therapeutic drug monitoring

## Abstract

**Background/Objectives**: Inflammatory bowel disease (IBD) management has evolved from conventional therapies to advanced biologics and targeted small molecules; however, clinical practice often relies on empirical treatment sequencing rather than individualized approaches. The heterogeneity of IBD phenotypes, variable treatment responses, and expanding therapeutic options necessitate a shift toward precision medicine. This review aims to synthesize current evidence on personalizing IBD therapy and provide an implementation framework for clinical practice. **Methods**: A narrative review was conducted encompassing peer-reviewed literature, recent network meta-analyses, and clinical guidelines. Evidence was gathered on treat-to-target strategies, therapeutic drug monitoring (TDM), clinical decision support systems, artificial intelligence applications, multi-omics platforms (genomics, transcriptomics, microbiome, metabolomics), advanced imaging modalities, and special populations including pediatric patients and pregnant women. **Results**: Treat-to-target strategies incorporating endoscopic and biochemical endpoints improve long-term outcomes when individualized to patient-disease factors. TDM-guided optimization enhances biologic efficacy and reduces immunogenicity. Emerging AI tools and multi-omics platforms show promise in predicting treatment response and patient stratification. Network meta-analyses provide comparative effectiveness estimates guiding advanced therapy selection in both Crohn’s disease and ulcerative colitis. Implementation of precision medicine frameworks remains constrained by regulatory, economic, and technical barriers. **Conclusions**: Personalizing IBD therapy through integration of precision medicine tools, patient-specific factors, and comparative effectiveness data represents the future of IBD management. Overcoming implementation barriers through standardized frameworks and multidisciplinary collaboration is essential to translate these advances into routine clinical practice.

## 1. Introduction

Inflammatory bowel disease (IBD) is a chronic, immune-mediated inflammatory disorder of the gastrointestinal tract comprising two principal entities: Crohn’s disease (CD) and ulcerative colitis (UC) [1,2]. CD is characterized by transmural, discontinuous inflammation that may involve any segment from the oral cavity to the anus, with a predilection for the terminal ileum, and is frequently complicated by strictures, fistulae, and abscesses [2]. UC is defined by continuous mucosal inflammation originating in the rectum and extending proximally in a contiguous pattern, confined exclusively to the colon [1]. Beyond the gut, IBD is associated with a broad spectrum of extraintestinal manifestations, neurological complications encompassing peripheral neuropathy, cerebrovascular events, and demyelinating disorders arise through immune-mediated, vascular, and nutritional mechanisms and contribute meaningfully to overall disease burden [3].

IBD affects over 6.8 million individuals worldwide, with the highest prevalence in North America and northern Europe [4]. While historically concentrated in Westernized nations, incidence is rising rapidly across Asia, the Middle East, and Latin America, reflecting the influence of environmental, dietary, and microbial exposures on genetic susceptibility [4]. The global healthcare burden is substantial; in the United States alone, direct costs exceed $30 billion annually, driven by hospitalization, biologic therapy expenditure, and surgical intervention, alongside considerable indirect costs from lost productivity and disability [4].

The pathogenesis of IBD involves a complex interaction among genetic susceptibility, immune dysregulation, gut microbiota dysbiosis, and environmental factors [1,2,5]. Genome-wide association studies have identified over 240 susceptibility loci including *NOD2*, *ATG16L1*, and *IL23R*—that disrupt epithelial barrier integrity and mucosal immune tolerance [5]. At the cellular level, bidirectional crosstalk between intestinal epithelial cells and the mucosal immune microenvironment sustains the chronic inflammatory cycle; notably, TNF-α–mediated suppression of IL-22/STAT3-dependent epithelial regeneration perpetuates barrier dysfunction [5]. Gut microbiota dysbiosis is characterized by depletion of protective commensals such as *Faecalibacterium prausnitzii* and expansion of pathobionts further amplifies mucosal immune activation [1]. Environmental triggers including Western dietary patterns, antibiotic exposure, and smoking modulate these pathways in genetically predisposed individuals [2].

The therapeutic approach to IBD has evolved substantially over recent decades. Conventional therapy encompassing aminosalicylates (5-ASA), corticosteroids, and immunomodulators (azathioprine, 6-mercaptopurine, methotrexate) remains the cornerstone of management for mild-to-moderate disease. In UC, 5-ASA agents are the primary treatment for mild-to-moderate activity; systemic corticosteroids are reserved for induction of remission in more active disease. In CD, budesonide is recommended for mild-to-moderate ileocaecal involvement, while systemic corticosteroids are used for moderate-to-severe cases [6,7]. However, conventional agents are limited by delayed therapeutic onset, long-term toxicity, and insufficient efficacy in refractory disease, with only approximately 30% of patients achieving sustained remission on conventional therapy alone [8].

**Figure 1:**
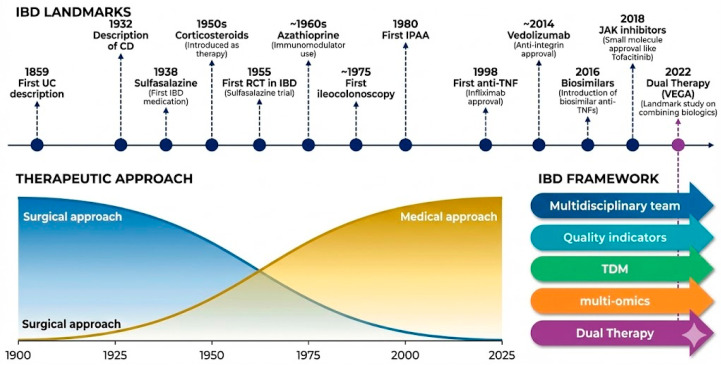
IBD framework.

Advanced therapies have transformed outcomes for patients with moderate-to-severe or refractory IBD. Biologic agents now span multiple distinct mechanisms of action anti-tumor necrosis factor (anti-TNF) agents (infliximab, adalimumab, certolizumab pegol), the gut-selective anti-integrin vedolizumab, the anti-IL-12/23 agent ustekinumab, and the selective anti-IL-23p19 agents risankizumab, mirikizumab, and guselkumab [6,7,9]. Targeted small molecules have further broadened the armamentarium, including the Janus kinase (JAK) inhibitors tofacitinib and upadacitinib, and the sphingosine-1-phosphate (S1P) receptor modulator etrasimod [6,7,9]. Emerging next-generation strategies including JAK1/2 PROTAC degraders that suppress JAK/STAT3-driven inflammation via the ubiquitin-proteasome pathway illustrate the continued mechanistic evolution of IBD therapeutics [10]. The historical evolution of IBD therapeutics and the contemporary precision medicine framework are illustrated in Figure 1. Despite these advances, response rates remain variable and clinicians must choose among multiple agents with differing efficacy, safety, and monitoring profiles [6,7,9].

Current real-world practice often resembles a one-size-fits-all approach: therapies are frequently sequenced empirically based on prior exposures, clinician preference, or safety considerations rather than individual molecular or clinical predictors of benefit [8]. This strategy carries important drawbacks, including delayed disease control, unnecessary exposure to ineffective treatments, avoidable adverse events, greater cumulative healthcare costs, and progression to irreversible bowel damage for patients who would have benefited from earlier aggressive therapy [8]. An “all-patient approach” demonstrates a ceiling of approximately 30% remission rates for currently available agents [8]. Moreover, the heterogeneity of IBD arising from the interplay between environment, genetics, microbiota, and the immune system means that no single treatment algorithm can adequately address the biological diversity of patients [1,2,5].

Taken together, these realities motivate a shift toward precision medicine approaches in IBD therapy, using clinical phenotypes, serologic and molecular biomarkers, endoscopic and imaging signatures, and emerging digital/dynamic markers (including multi-omics and AI-derived models) to predict disease course and match patients to the therapy most likely to induce durable remission while minimizing harm. Achieving this goal requires synthesizing evidence on predictive biomarkers, comparative effectiveness, safety trade-offs. Current landscape of diagnostic and therapeutic tools was summarized, appraising the evidence for biomarker-guided treatment decisions, and propose a practical framework for translating precision approaches into routine IBD care.

## 2. Methods

A narrative review methodology was employed rather than a systematic review approach, given the broad and heterogeneous scope of this work, which spans multiple domains including precision biomarkers, therapeutic drug monitoring, multi-omics platforms, artificial intelligence, comparative effectiveness, and implementation frameworks domains that are not amenable to a single unified search strategy or PRISMA-style synthesis.

A literature search was conducted across PubMed/MEDLINE, EMBASE, and the Cochrane Library, covering publications from 2000 to 2025, with priority given to publications from 2015, reflecting the emergence of advanced biologics and precision medicine tools in IBD. Search terms included combinations of: “inflammatory bowel disease,” “Crohn’s disease,” “ulcerative colitis,” “precision medicine,” “personalized therapy,” “therapeutic drug monitoring,” “artificial intelligence,” “multi-omics,” “biomarker,” and “treat-to-target.”

Overall, approximately 2754 records were identified, of which 30 studies were included after title/abstract screening and full-text review. Study selection was guided by relevance to predefined thematic domains (precision biomarkers, therapeutics, AI, and implementation frameworks). Inclusion criteria comprised peer-reviewed English-language publications including randomized controlled trials (RCTs), network meta-analyses (NMAs), prospective cohort studies, and major clinical practice guidelines (AGA, ECCO, STRIDE). RCTs and NMAs were considered primary sources; clinical practice guidelines provided consensus-level recommendations; observational and real-world studies were incorporated where higher-level evidence was limited. This review synthesizes findings from existing published NMAs rather than performing a new independent comparative analysis.

## 3. Principle of Individualizing Therapy

The evolution of IBD management has been shaped by the concept of “treat-to-target”, which is a management strategy in which predefined therapeutic endpoints (clinical, endoscopic, biochemical, and histologic) are established prospectively, and treatment is escalated or modified until those targets are achieved, was first formalized in the STRIDE-I position statement, which defined therapeutic targets to guide clinical decision-making [11]. Since then, the rapid introduction of novel biologics and small molecules has expanded both the potential and complexity of treatment, enabling goals beyond conventional symptom control. The updated STRIDE-II recommendations establish hierarchical treatment goals stratified by disease type and timeline [12]. For UC, short-term targets (3–6 months) include clinical remission combined with endoscopic healing, defined as Mayo endoscopic subscore ≤ 1. Long-term targets extend to histologic remission, as persistent microscopic inflammation despite endoscopically healed mucosa predicts higher relapse rates. In CD, short-term goals prioritize clinical remission plus endoscopic response (>50% reduction in ulceration) or remission (absence of ulceration), while long-term objectives include transmural healing documented by cross-sectional imaging, particularly in patients with penetrating complications.

Beyond endoscopy, STRIDE-II endorses normalization of inflammatory biomarkers as intermediate targets. Fecal calprotectin levels below 150 μg/g correlate strongly with endoscopic remission, while persistent elevation predicts disease relapse. The updated recommendations emphasize patient-reported outcomes as complementary targets, acknowledging that endoscopic healing without functional improvement represents incomplete therapeutic success. Contemporary management increasingly emphasizes individualized treatment strategies, integrating patient-specific disease characteristics, treatment response, and risk profiles into the therapeutic algorithm to optimize outcomes [12].

### Patient-Reported Outcomes and Shared Decision-Making

Patient-reported outcomes (PROs) is a formal initiative to recognize complementary treatment targets clinical, endoscopic, and biochemical measures, representing a shift from physician-centered to patient-centered care [12]. PROs capture disease impact from the patient perspective, including symptoms, functional status, and health-related quality of life. In UC, PRO2 measures rectal bleeding and stool frequency; scores below 1 predict endoscopic remission with high specificity: a PRO2 score < 1 identifies endoscopic remission in UC with 94.9% specificity and 86.4% positive predictive value [13,14].

Shared decision-making (SDM) represents the cornerstone of truly personalized medicine, integrating clinical evidence with patient values, preferences, and individual circumstances to arrive at optimal therapeutic decisions [15,16]. The majority of IBD patients consider active involvement in treatment decisions as very important, with patient involvement in SDM correlating significantly with higher treatment satisfaction, improved medication adherence, and ultimately better clinical outcomes. The PROSPECT tool (Personalized Risk and Outcome Prediction Tool) generates individualized risk prediction according to clinical and serologic variables for CD complications [17]. A multicenter randomized trial evaluating an SDM program with PROSPECT demonstrated significantly higher rates of appropriate early intensive therapy adoption compared to standard care, with improved patterns of medication use and clinical outcomes [17].

## 4. Patient Disease-Related Factors

Patient factors contribute significantly to the concept of individualized therapy in IBD, as demonstrated by the landmark IBSEN study, which followed (519) UC patients over 20 years in a population-based inception cohort. This comprehensive study revealed substantial heterogeneity in disease outcomes based on individual patient characteristics, supporting the need for personalized treatment approaches rather than uniform therapeutic strategies. Disease extent at diagnosis emerged as a critical prognostic factor.

Age at diagnosis also significantly influenced outcomes, with younger patients (<40 years) demonstrating increased risks of hospitalization (OR = 3.7) and colectomy, suggesting that age-specific treatment intensification may be warranted.

The IBSEN study further demonstrated that early mucosal healing at one year was independently associated with dramatically improved long-term outcomes, including 60% lower colectomy risk (HR = 0.4) and 50% reduced relapse rates (HR = 0.5), emphasizing the importance of individualized treatment targets and monitoring strategies. Patient-reported disease courses varied substantially, with 69% experiencing initial high activity followed by remission, while others had chronic continuous (8%) or intermittent symptoms (19%), reflecting diverse pathophysiological patterns that may require tailored therapeutic approaches [18].

### 4.1. Pediatric Consideration in IBD

Pediatric IBD populations require modification for age-specific factors including growth optimization, risk stratification, and transition to adult care. The RISK cohort study identified genetic (*NOD2*, *ATG16L1*), serologic (ASCA, anti-CBir1), and clinical predictors that forecast complicated disease course with 78% accuracy, enabling precision-based risk stratification at diagnosis [19]. High-risk children warrant accelerated treatment algorithms with early biologic therapy, while lower-risk patients may be suitable for conventional step-up approaches.

### 4.2. Growth as a Treatment Target

Growth impairment represents a unique pediatric complication, with inflammatory cytokines suppressing the growth hormone-IGF-1 axis during critical developmental periods [20]. The REACH trial demonstrated that infliximab achieved 88% clinical response and 59% remission with significant improvements in height velocity Z-scores [21], while the IMAgINE-1 trial established adalimumab efficacy with 38.8% remission and documented growth improvement [22]. Early anti-TNF therapy within 90 days of diagnosis demonstrates superior effectiveness in high-risk pediatric patients, achieving sustained steroid-free remission and catch-up growth [19].

### 4.3. Transition to Adult Care

The transition from pediatric to adult IBD care represents a vulnerable period associated with medication nonadherence, poor follow-up, and disease relapse. Structured transition programs improve outcomes by gradually shifting responsibility from parents to patients over several years rather than abrupt transfer at age 18. The ImproveCareNow quality improvement network demonstrated that structured transition programs initiated by age 12 improve clinical outcomes, reduce corticosteroid use, and increase sustained remission rates [23,24]. STRIDE targets remain consistent across transition, but implementation requires adaptation to psychosocial realities of emerging adulthood.

### 4.4. Pregnancy Consideration in IBD

Managing IBD during pregnancy requires balancing maternal disease control, which directly impacts fetal outcomes, against potential medication risks. Current evidence strongly supports maintaining remission throughout pregnancy, as active disease carries greater risks to both mother and fetus than most IBD medications, including increased rates of preterm birth (OR 2.1), low birth weight (OR 2.3), spontaneous abortion, and cesarean delivery [25].

The prospective PIANO Registry, encompassing over 1800 completed pregnancies, represents the largest and most comprehensive study of medication safety during pregnancy in IBD patients [25]. This landmark study demonstrated no increased risk of congenital abnormalities with thiopurine exposure (adjusted OR 1.0, 95% CI 0.5–2.1) or anti-TNF therapy (adjusted OR 0.9, 95% CI 0.5–1.6) compared to unexposed controls, providing definitive reassurance about first-trimester safety of these agents. Combination therapy with anti-TNF plus thiopurines showed similar safety profiles without additional fetal risk, contradicting earlier theoretical concerns about dual immunosuppression. However, combination-exposed infants demonstrated slightly increased infection rates in the first year of life (adjusted OR 1.4, 95% CI 1.0–1.9), attributable primarily to minor respiratory and gastrointestinal infections rather than serious opportunistic infections. Additionally, PIANO showed no increased complications with ustekinumab or vedolizumab exposure compared to anti-TNF agents, supporting continuation of these biologics throughout pregnancy to maintain disease remission [25]. Tofacitinib demonstrated fetal toxicity and teratogenicity in animal studies at exposures exceeding standard human doses, leading to current recommendations to avoid JAK inhibitors during pregnancy [26].

## 5. Tools to Guide Individualization

### 5.1. Therapeutic Drug Monitoring (TDM) and Clinical Decision

The translation of precision medicine principles into clinical practice requires validated tools that can predict treatment responses and guide therapeutic decisions throughout the disease course. Therapeutic drug monitoring (TDM) has emerged as one such foundational tool for personalizing biologic therapy in IBD, operating by measuring trough drug concentrations and detecting anti-drug antibodies (ADAs) to guide dose adjustment, prevent treatment failure, and optimize pharmacokinetic outcomes in individual patients [27]. While biological therapies have substantially improved clinical outcomes, rigorous monitoring and therapy optimization remain essential to maximize effectiveness and minimize adverse events. TDM enhances treatment outcomes in a cost-effective manner by measuring drug trough concentrations and detecting anti-drug antibodies (ADAs), allowing clinicians to tailor therapy according to individual pharmacokinetic variability, disease activity, and mechanisms of treatment failure. Although most evidence has focused on biologic monotherapy, emerging data indicate that TDM is equally relevant in combination therapy, supporting optimized dosing strategies and improved durability of response [28].

Beyond TDM, Clinical decision support tools (CDSTs) integrate clinical, biochemical, pharmacokinetic, and sometimes genomic or imaging data to support evidence-based treatment decisions at the point of care. CDSTs can guide biologic selection, predict treatment response, optimize dosing strategies, and identify patients at risk of non-response or early loss of response. VDZ-CDSTs showed strong discrimination across clinical and endoscopic outcomes, allowing for more accurate identification of patients likely to achieve remission or maintain treatment persistence without the need for dose escalation or surgery [29]. Importantly, the substantial agreement (73.7%) between UST- and VDZ-CDSTs suggests potential cross-biologic predictive value, supporting the broader applicability of CDST-guided precision therapy [29].

### 5.2. Artificial Intelligence

Artificial intelligence (AI) is emerging as a powerful tool of individualized medicine in IBD, encompassing a range of model types including convolutional neural networks for endoscopic image analysis, machine learning algorithms for treatment response prediction, and recurrent neural networks for relapse risk forecasting, applied across diverse clinical and molecular data sources. By integrating endoscopic and radiologic imaging, biochemical biomarkers, clinical records, and multi-omics datasets such as genomics, transcriptomics, proteomics, and microbiome profiles, these AI-driven models can identify complex patterns not detectable by human assessment alone [30,31]. These capabilities allow AI-driven models to improve diagnostic precision, standardize endoscopic scoring, and monitor disease activity with high accuracy [32]. More importantly, AI facilitates precision therapeutics by predicting patient-specific responses to biologic and small-molecule therapies, distinguishing likely responders from non-responders, and forecasting relapse risk [33]. Machine learning algorithms can integrate clinical, genomic, transcriptomic, proteomic, and microbiome data to achieve area under the curve (AUC) values ranging from 0.77 to 0.85 for predicting treatment responses to various biologic therapies [34,35]. This individualized approach reduces trial-and-error treatment selection, shortens time to remission, and optimizes the use of high-cost medications [36].

Emerging wearable technologies integrated with AI enable continuous monitoring of inflammatory biomarkers such as C-reactive protein, IL-6, and fecal calprotectin, allowing for dynamic disease tracking and early intervention before clinical flares [37]. These digital health platforms demonstrate feasibility in reducing hospitalizations and outpatient visits while maintaining quality of care through personalized risk stratification [36].

Collectively, AI serves as a critical tool for personalized IBD care, supporting treatment decisions that reflect each patient’s unique biological, clinical, and molecular profile [38]. The integration of AI-enabled endo-histo-omics approaches, combining endoscopic imaging, histological analysis, and multi-omic data, represents the next in precision IBD medicine, enabling unprecedented patient characterization and therapeutic personalization. Despite these promising results, most AI models are derived from retrospective, single-center datasets, limiting generalizability, and absence of prospective clinical trials remain major barriers to routine clinical implementation.

### 5.3. Multi Omics Platforms (Genomics, Microbiome, Transcriptomics)

Multi-omics platforms integrating genomics, transcriptomics, metabolomics, and microbiome profiling have emerged as transformative tools for addressing the heterogeneity of IBD [35], serving two interconnected clinical roles: (1) biomarker discovery identifying transcriptomic signatures, metabolomic profiles, and microbial markers associated with disease phenotype, complication risk, and drug response; and (2) patient stratification classifying patients into biologically distinct subgroups to guide individualized treatment selection, ranging from TPMT/NUDT15 pharmacogenomic testing for thiopurine dosing to transcriptomic IBD1/IBD2 profiling in the PROFILE trial. These technologies thereby enable clinicians to move beyond clinical phenotype alone toward molecularly informed therapeutic decisions.

Pharmacogenomic testing represents the most clinically mature application of precision medicine in IBD. Genotyping for thiopurine methyltransferase (TPMT) and nudix hydrolase 15 (NUDT15) variants is currently the only genomic test recommended by major clinical practice guidelines for IBD management [39]. These genes encode enzymes responsible for thiopurine drug metabolism, and loss-of-function variants significantly increase the risk of severe, potentially life-threatening myelosuppression when patients receive standard-dose azathioprine or mercaptopurine.

Implementation of TPMT and NUDT15 testing has demonstrated clinical utility across diverse populations. In a large tertiary hospital experience, 13% of tested patients carried actionable variants requiring dose modifications, with NUDT15 testing identifying an additional 3.9% of at-risk patients beyond those detected by TPMT testing alone [40]. This finding is particularly relevant for Asian and Hispanic populations, where NUDT15 variant alleles are more prevalent [40].

Gene expression in intestinal tissues has identified transcriptomic signatures distinguishing treatment responders from non-responders prior to therapy initiation. Pathway analyses reveal that responders exhibit baseline expression patterns in immune signaling cascades, ECM remodeling pathways, and epithelial barrier function genes compared to non-responders [39].

Metabolomic profiling, particularly of serum and fecal bile acids, sphingolipids, and amino acid metabolites, provides predictive information [40]. The integration of metabolomic data with microbiome profiles is especially powerful, as many metabolites are products of host-microbial co-metabolism, directly linking microbial community function to host physiology and drug response [41]. Gut microbiome composition correlates strongly with specific IBD phenotypes and complications. Metagenomics has revealed distinct microbial signatures associated with stricturing versus penetrating CD, providing mechanistic insights and potential predictive biomarkers [42]. In UC, metagenomic profiles can predict post-surgical complications. The presence of certain pathogenic species and depletion of beneficial microbiota may predict pouchitis risk following ileal pouch–anal anastomosis surgery [43].

The PROFILE trial represents a milestone in stratified medicine for IBD. This biomarker-driven, RCT used a whole-blood transcriptomic signature (IBD1/IBD2) to classify newly diagnosed CD patients into high- or low-risk phenotypes, aiming to personalize early treatment strategies. Although the study did not demonstrate a statistically significant benefit of a biomarker-guided approach over standard care, it provided the first prospective evidence that transcriptomic profiling can feasibly stratify patients at diagnosis and guide therapeutic decisions [44,45]. Importantly, a clear distinction must be made between clinically actionable tools (e.g., TPMT/NUDT15 testing) and emerging multi-omic approaches, which remain investigational and require further validation before integration into routine care.

### 5.4. Intestinal Ultrasound and Advanced Imaging

Advanced imaging modalities have emerged as essential tools for personalized IBD management, enabling non-invasive assessment of disease activity, treatment response monitoring, and prediction of clinical outcomes. These technologies complement endoscopic evaluation while reducing procedural burden and providing transmural information [46].

Intestinal Ultrasound (IUS) is an excellent tool for the assessment of suspected IBD, with a very high negative predictive value. It accurately assesses disease activity, disease complications, and in the pre-treatment phase, provides a benchmark for subsequent follow-up [47]. IUS provides real-time, non-invasive assessment of bowel wall thickness, vascularity, and transmural inflammation without radiation exposure or sedation, making it ideal for serial monitoring during therapy optimization. Advanced imaging provides prognostic stratification that personalizes treatment intensity from diagnosis, with baseline MRE features predicting disease course independent of clinical factors. In the VERSIFY trial MRE sub study, standardized MRE protocols demonstrated feasibility of assessing transmural inflammation features including bowel wall edema, wall thickness, and enlarged lymph nodes in patients receiving vedolizumab, with progressive improvement in these parameters observed through week 52 [47].

The key tools currently available to support precision medicine in IBD, along with their levels of evidence and guideline recommendations, are summarized in Table 1.

## 6. Comparative Effectiveness of Advanced Therapies

The expansion of therapeutic options in IBD necessitates comparative effectiveness data to guide treatment selection. Network meta-analyses informing the 2024–2025 American Gastroenterological Association (AGA) clinical guidelines provide the most comprehensive evidence for positioning biologics and small molecules across different clinical scenarios, stratified by prior treatment exposure [48,49,50].

### 6.1. Crohn’s Disease

#### 6.1.1. First-Line Advanced Therapy in Biologic-Naïve CD

In patients with moderate-to-severe CD who have not received prior biologic therapy, moderate-to-high certainty evidence supports infliximab, adalimumab, vedolizumab, ustekinumab, risankizumab, mirikizumab, and guselkumab for inducing clinical remission, with these agents demonstrating comparable efficacy when used as first-line therapies [48]. The updated 2025 AGA guidelines suggest early use of these higher-efficacy advanced therapies compared to traditional step-up approaches using corticosteroids or immunomodulators, representing shift toward more aggressive initial management to prevent disease progression [48].

#### 6.1.2. Therapeutic Sequencing After Biologic Failure in CD

Among patients with CD previously exposed to anti-TNF therapy, moderate-to-high certainty evidence supports adalimumab, ustekinumab, risankizumab, guselkumab, and upadacitinib for inducing remission, with active comparator analyses showing moderate certainty evidence favoring risankizumab and guselkumab over vedolizumab and ustekinumab [49].

#### 6.1.3. Endoscopic Outcomes as Treatment Targets

Network meta-analysis specifically evaluating endoscopic endpoints in CD demonstrated that JAK1 inhibitors and anti-IL23p19 agents may be the most effective among non-TNF-targeting therapies for inducing endoscopic response, with these findings informing treat-to-target strategies that prioritize mucosal healing [50]. The emphasis on endoscopic and histologic healing reflects evolving therapeutic goals beyond symptom control alone.

### 6.2. Ulcerative Colitis

#### 6.2.1. First-Line Advanced Therapy in Biologic-Naïve UC

Network meta-analysis of 27 induction trials and 18 maintenance trials demonstrated that, among biologic-naïve patients, infliximab, adalimumab, vedolizumab, ustekinumab, tofacitinib, upadacitinib, mirikizumab, guselkumab, and etrasimod achieved significantly higher rates of clinical remission compared to placebo [49].

#### 6.2.2. Therapeutic Sequencing After Biologic Failure in UC

In UC patients who failed anti-TNF therapy, moderate-to-high certainty evidence demonstrated superior efficacy for upadacitinib, tofacitinib, vedolizumab, ustekinumab, and guselkumab compared to placebo, with upadacitinib showing advantages over several other agents in direct network comparisons [49]. These findings support mechanism-of-action switching strategies rather than cycling within the same therapeutic class after primary non-response.

## 7. Future Direction

Primary non-response to biologic therapies occurs in 30–40% of IBD patients, and secondary loss of response develops in an additional 10–50% over time, representing the central unmet need that precision medicine must address [51]. The PROFILE trial, while not demonstrating superiority of biomarker-guided therapy over standard care, reinforced the need for refined biomarkers and integrated multi-omic approaches in future precision medicine frameworks. Several priorities emerge from this review:

First, prospective validation of AI predictive models in diverse real-world populations is urgently needed, as most published models were derived from single-center academic cohorts. Second, development of multi-omic composite biomarker panels with regulatory-grade analytical validation represents a critical next step. Third, head-to-head prospective trials comparing biomarker-guided versus empiric treatment strategies are needed to establish clinical utility and cost-effectiveness. Forth, global equity in access must be addressed, as precision medicine tools remain largely restricted to academic centers in high-income countries. This review has several limitations. First, as a narrative review, the literature search was not conducted using a systematic PRISMA-based methodology, introducing the possibility of selection bias. Second, most evidence cited derives from academic medical centers and clinical trial populations, which may not reflect real-world IBD patients across diverse healthcare settings. Third, many of the discussed precision tools, particularly AI models and multi-omic panels, remain investigational and have not undergone prospective validation, limiting the strength of recommendations.

## 8. Practical Implementation Framework

Translating precision medicine from research to routine IBD practice requires addressing implementation barriers that extend beyond biomarker validation. Success depends on navigating regulatory pathways most notably the absence of established approval frameworks for AI-based clinical decision support tools and multi-omic companion diagnostics in most jurisdictions, establishing sustainable economic models, and ensuring equitable access across diverse healthcare settings.

### 8.1. Phased Integration Strategy

**Figure 2:**
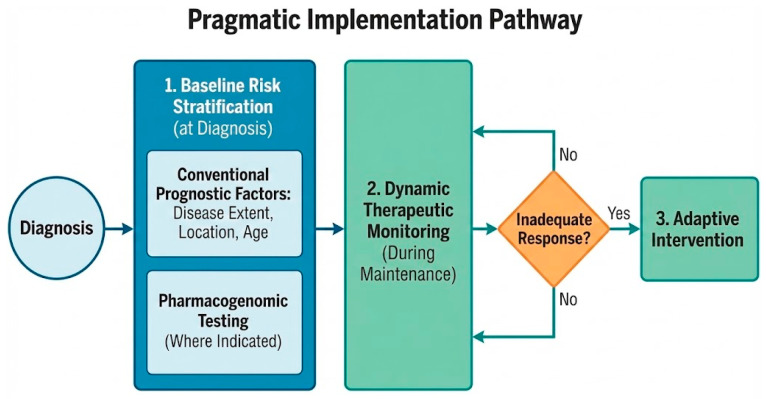
Pragmatic implementation pathway.

A pragmatic implementation pathway should progress sequentially: baseline risk stratification at diagnosis, dynamic therapeutic monitoring during maintenance, and adaptive intervention when response proves inadequate. The baseline assessment builds upon conventional prognostic factors such as disease extent, location, and age at diagnosis, while incorporating pharmacogenomic testing where indicated Figure 2.

During maintenance therapy, the emphasis shifts to proactive monitoring strategies that prevent disease progression rather than reacting to clinical deterioration. When therapeutic decisions arise, integrated decision support platforms can synthesize multiple data streams to guide biologic selection or switching strategies.

### 8.2. Overcoming Adoption Barriers

Economic sustainability represents a critical challenge for precision medicine adoption. While biologics consume substantially, precision approaches that improve first-line selection may ultimately reduce costs by decreasing treatment failures and disease complications. Strategic deployment of biosimilars creates financial flexibility to support biomarker testing framework. However, sustained implementation requires demonstrating value through population-level outcome improvements and favorable cost-effectiveness compared to empiric treatment sequencing [52].

Beyond economics, technical framework demands attention. Integrating laboratory systems, electronic health records, and clinical decision platforms requires standardized data and institutional investment. Workforce capacity building remains equally essential as clinicians need competency in interpreting genomic results, applying pharmacokinetic principles, and utilizing decision support tools effectively. Implementation strategies must ensure validation studies include diverse populations and that access barriers do not restrict precision medicine to academic medical centers.

Successful implementation ultimately requires cross-stakeholder collaboration among clinicians, researchers, and patient advocates to establish validation standards, streamline approval processes, and align with outcomes rather than procedural volume. 

## 9. Conclusions

Personalized IBD therapy has transitioned from concept to an actionable clinical framework. Among currently available precision tools, TPMT/NUDT15 pharmacogenomic testing, proactive TDM, clinical decision support tools, and validated risk stratification models represent the most promising candidates for near-term clinical adoption. They are guideline-supported, technically feasible in most centers, and have demonstrated measurable impact on patient outcomes. In contrast, AI-driven multi-omic integration models, while promising with early AUC values of 0.77–0.85 for treatment response prediction, require prospective validation in diverse populations and regulatory approval before widespread adoption. The PROFILE trial serves as an important reminder that biomarker discovery alone is insufficient without demonstrated clinical utility. Bridging this gap requires not only scientific progress but also coordinated efforts in regulatory approval, health system investment, and clinician training.

## Figures and Tables

**Table 1:** Precision medicine tools in IBD: evidence level.

Precision Medicine Tool	Evidence Base	Guideline Recommendation
TPMT/NUDT15 Pharmacogenomics	Prospective cohorts	AGA, ECCO
TDM	Multiple RCTs and prospective studies; cost-effective optimization of biologic dosing	AGA, ECCO
PROs	RCTs and validation studies; PRO2 score: 94.9% specificity for endoscopic remission in UC	STRIDE-II
IUS	Prospective cohorts; high NPV for IBD assessment; VERSIFY MRE sub-study	ECCO
CDSTs	Retrospective cohort studies; VDZ-CDST and UST-CDST demonstrate 73.7% cross-biologic agreement	Require prospective multicentre validation
AI Predictive Models	Single-center observational studies; AUC 0.77–0.85 for treatment response prediction; CNN-based endoscopic scoring	Investigational
Multi-Omics Platforms (genomics, transcriptomics, metabolomics, microbiome)	Exploratory cohort studies; PROFILE RCT (IBD1/IBD2 transcriptomic stratification); multi-omic integration analyses	Investigational

Abbreviations: AGA, American Gastroenterological Association; CDST, clinical decision support tool; ECCO, European Crohn’s and Colitis Organisation; CNN, convolutional neural network; IBD, inflammatory bowel disease; IUS, intestinal ultrasound; NPV, negative predictive value; NUDT15, nudix hydrolase 15; PRO, patient-reported outcome; RCT, randomized controlled trial; STRIDE, Selecting Therapeutic Targets in Inflammatory Bowel Disease; TDM, therapeutic drug monitoring.

## Data Availability

No new data were created or analyzed in this study.

## Abbreviations

The following abbreviations are used in this manuscript:

IBD	Inflammatory Bowel Disease
UC	Ulcerative Colitis
CD	Crohn’s Disease
5-ASA	5-Aminosalicylic Acid
TNF	Tumor Necrosis Factor
JAK	Janus Kinase
STRIDE	Selecting Therapeutic Targets in Inflammatory Bowel Disease
PRO	Patient-Reported Outcome
S1P	Sphingosine-1-Phosphate
SDM	Shared Decision-Making
TDM	Therapeutic Drug Monitoring
ADA	Anti-Drug Antibody
CDST	Clinical Decision Support Tool
AI	Artificial Intelligence
AUC	Area Under the Curve
TPMT	Thiopurine Methyltransferase
NUDT15	Nudix Hydrolase 15
MRE	Magnetic Resonance Enterography
IUS	Intestinal Ultrasound
AGA	American Gastroenterological Association
IPAA	Ileal Pouch–Anal Anastomosis
IGF-1	Insulin-like Growth Factor 1
PIANO	Pregnancy in Inflammatory and Autoimmune Conditions
RCT	Randomized Controlled Trial
